# Retrospective Analysis of Adoptive TIL Therapy plus Anti-PD1 Therapy in Patients with Chemotherapy-Resistant Metastatic Osteosarcoma

**DOI:** 10.1155/2020/7890985

**Published:** 2020-10-01

**Authors:** Xiang Zhou, Junlong Wu, Chunguang Duan, Yingjie Liu

**Affiliations:** ^1^Department of Orthopedic Surgery, Luoyang Central Hospital Affiliated to Zhengzhou University, Luoyang 471009, China; ^2^Department of Orthopedic Surgery, Shenzhen University General Hospital, Shenzhen 518055, China

## Abstract

**Background:**

The pathological subtype of osteosarcoma is one of the most common malignant bone tumors. Notably, chemotherapy-resistant metastatic osteosarcoma has been reported to cause significant mortality and shows poor prognosis with the currently available multidisciplinary treatments. This study investigated whether combined adoptive TIL and anti-PD1 therapy improves the prognosis of patients with chemotherapy-resistant metastatic osteosarcoma.

**Methods:**

A total of 60 patients with chemotherapy-resistant metastatic osteosarcoma between June 2016 and March 2018 were enrolled. The primary endpoint was to evaluate the safety and adverse effects (AEs) of infusions of TIL and anti-PD1 therapy in the patients. Besides, secondary endpoints included assessing the objective response rate (ORR), progression-free survival time (PFS), and overall survival time (OS).

**Results:**

We reported that combined TIL therapy and anti-PD1 therapy is safe and all treatment-related AEs were reversible or manageable. The ORR of all the patients is 36.67%, and patients with more infusions of TIL and CD8^+^TIL, less infusions of CD8^+^PD1^+^TIL, and less infusion of CD4^+^FoxP3^+^TIL exhibited increased PFS and OS.

**Conclusion:**

This study determined that combined TIL and anti-PD1 therapy is safe and effective in metastatic osteosarcoma patients with chemotherapy resistance.

## 1. Introduction

Osteosarcoma is mortal cancer predominantly affecting children and young adults with a peak age of about 20 years [1]. Approximately 70% of patients with nonmetastatic osteosarcoma can survive for a long time when subjected to the currently developed multidisciplinary treatments [2, 3]. However, progress has slowed over the past 30 years, and efforts to improve outcomes with intensifying chemotherapy regimens or adding novel nonselective agents are unsuccessful [4–7]. Moreover, about 25-30% of osteosarcoma patients present with clinical metastases at the time of the first diagnosis, and patients without clinical metastases at initial presentation often develop metastatic disease despite undertaking the multidisciplinary treatments [8, 9]. Of note, chemotherapy is the main treatment method for these patients either with or without surgery; however, it is not effective against metastatic osteosarcoma with 5-year overall survival time (OS) less than 20% [2, 3]. Additionally, patients with metastatic osteosarcoma rapidly develop more lesions and become resistant to chemotherapy. Therefore, new therapeutic strategies for metastatic osteosarcoma, particularly for patients exhibiting chemotherapy resistance, are urgently needed to improve the prognosis.

Recent studies have reported immune checkpoint inhibitors, particularly those that block the PD1/PDL1 pathway; this indicates remarkable clinical success in many cancer types including osteosarcoma [10–14]. However, this form of immunotherapy has vastly changed the treatment landscape and achieved FDA approval for osteosarcoma [15]. Besides, most patients have a limited objective response rate (ORR) to these drugs, indicating that in-depth research should be conducted to understand the immunocompetency of the patients. A multicenter, phase 2 trial of patients from the Sarcoma Alliance for Research through Collaboration (SARC028) studied pembrolizumab in patients (12 years or older) with advanced soft tissue and bone sarcoma. Notably, ORR was achieved in 1 out of 22 (5%) patients with osteosarcoma [11]. This may have been attributed to the effects of these drugs which depend on preexisting endogenous antitumor immune responses. In many settings, cancer patients generate T cell immune responses against tumors in the microenvironment, and tumor-reactive cytotoxic T lymphocytes (CTLs) infiltrate the tumor thereby inhibiting or eliminating the tumor [16, 17]. However, many studies suggest that CTLs are induced during metastatic osteosarcoma progression but are later exhausted in the tumor microenvironment [18–20]. Of note, the ORR of anti-PD1 therapy is slightly dependent on the numbers of TILs in the microenvironment [21, 22]. Therefore, anti-PD1 therapy alone may not be an effective treatment strategy for metastatic osteosarcoma.

Adoptive cell therapy (ACT) of tumor-infiltrating lymphocytes (TILs) has achieved a satisfactory treatment effect for metastatic melanoma patients reporting ORR of between 40 and 70% [23–26]. However, TILs represent a potential therapeutic approach in numerous malignant pathologies, yet there are no reports on its underlying mechanism against osteosarcoma [27–30]. A report from a preclinical study indicated that TILs extracted from osteosarcoma could penetrate the tumor microenvironment and showed cytotoxic effects against allogeneic tumor cells; this demonstrates that TIL therapy could be an efficient strategy for treating osteosarcoma [31]. Anti-PD1 therapy relies on TILs in the tumor microenvironment; therefore, combined anti-PD1 therapy and TILs may induce potential antitumor effects on metastatic osteosarcoma patients.

The study is primarily aimed at assessing the response of combined adoptive TIL therapy and anti-PD1 therapy in patients with chemotherapy-resistant metastatic osteosarcoma. Also, it sought to determine whether biomarkers that predict response to TIL therapy and anti-PD1 therapy could be identified from cultured TILs. This will help in identifying patients most likely to benefit from the newly proposed therapy.

## 2. Materials and Methods

### 2.1. Patients

Sixty patients with a clinical diagnosis of metastatic osteosarcoma were enrolled in this study. In addition, the study group had experienced disease progression after chemotherapy, exhibiting chemotherapy resistance. We followed the methods of Chen et al. for the inclusion and exclusion [32]. Other inclusion criteria included (1) discontinuing any cancer therapy before enrollment, (2) having age above 11 years, (3) life expectancy of more than 3 months, (4) Eastern Cooperative Oncology Group (ECOG) performance status of 0-1, (5) adequate organ function, and (6) lesions that can be assessed using the standard response evaluation criteria in solid tumors (RECIST 1.0 version 1.1) guidelines [33]. The following exclusion criteria were applied: previous treatment with anti-CTLA4 or anti-PD1/PDL1 therapy, any form of primary immunodeficiency or history of autoimmune diseases, ongoing systemic infections and concurrent systemic steroid therapy, and recruitment into other clinical trials. All participating patients provided informed consent.

### 2.2. Study Design and Procedures

This single-center clinical study was approved by the Ethics Committee at the Affiliated Luoyang Central Hospital of Zhengzhou University. All methods and procedures associated with this study were conducted in accordance with the Good Clinical Practice guidelines and accorded ethically with the principles of the Declaration of Helsinki and local laws. All authors had access to the study data and reviewed and approved the final manuscript. Infusions of anti-PD1 therapy (nivolumab, 3 mg/kg) were administered to the patients at our department for two weeks for one cycle. All patients received at least 8 cycles of infusions or received cycles until they experienced disease progression or unacceptable adverse effects (AEs) or withdrew from this study. In the first cycles of anti-PD1 therapy, TILs were transfused into patients. Patients with disease progression were received multidisciplinary synthetic therapy and best support care. After treatment, all the patients were received follow-up to examine the tumor status every 3 months. The follow-up deadline was February 2020.

### 2.3. Outcome Measures

The primary endpoint was to evaluate the safety and AEs of infusions of TILs plus nivolumab in the patients. Secondary endpoints included assessments of the objective response rate (ORR), progression-free survival time (PFS), and overall survival time (OS). Safety evaluations primarily consisted of clinical and laboratory abnormalities that were monitored throughout the study up until two weeks after the last infusion of nivolumab. AEs were evaluated using the National Cancer Institute Common Toxicity Criteria version 4.0 [34]. Treatment-associated AEs were assessed during the treatment and observation periods, and the highest observed grade was recorded for each patient. In each patient, lesions were evaluated using computed tomography (CT) or magnetic resonance imaging (MRI) every 3 months. The ORR were assessed by RECIST version 1.1 [33]. Potential prognostic factors were also analyzed by univariate and multivariate analyses based on combined TILs and anti-PD1 therapy. The PFS was calculated from the date of immunotherapy to the time of disease progression. Patients free of these events were censored at the time of the last contact. The OS was calculated from the date of immunotherapy to the time of death, and patients who were alive at the time of the last contact were censored. PFS and OS were calculated by the Kaplan-Meier method.

### 2.4. Generation of TILs

Fresh tumor tissues from metastatic sites were obtained from each patient by thick needle puncture and culture of the TILs. The tumor tissues were confirmed by two independent pathologists in our hospital before culturing the TILs. The detailed protocol used is similar to the previously described procedure [23, 35, 36], as follows: [1] Tumor tissues were sliced into pieces of about 2 to 3 mm^3^ in size using a scalpel. [2] Collagenase type IV (Sigma-Aldrich, St. Louis, MO, USA, 1 mg/mL), DNase I (Sigma-Aldrich, St. Louis, MO, USA, 2 U/mL), and hyaluronidase type V (Sigma-Aldrich, St. Louis, MO, USA, 0.5 U/mL) were used to digest the tissues for approximately 3 hours at room temperature to obtain single-cell suspensions. [3] The single-cell suspensions were filtered, washed twice with phosphate-buffered saline (PBS), and incubated in a 12-well plate at a concentration of 1.0 × 10^6^ TILs/mL in X-VIVO medium (Muenchensteinerstrasse 38, CH-4002 Basel, Switzerland) with 7000 IU/mL recombinant human interleukin-2 (rhIL-2, Novartis, UK). This day was considered day 0. [4] On the 1^st^ day, the cell suspensions were removed and further purified via Ficoll gradient. The purified bulk TIL culture was maintained at a concentration of 1‐2 × 10^6^ cells/mL in X-VIVO medium with 7000 IU/mL rhIL-2 until all other cells (including osteosarcoma cells) were eliminated to achieve a cell number of at least 5 × 10^7^ TIL cells. The culturing process occurred for approximately 10 to 14 days. [5] Eventually, the cultured TIL cells were immediately used with anti-CD3 antibody (GE Healthcare Biosciences, Pittsburgh, PA, USA; 30 ng/mL) and 1000 IU/mL rhIL-2 for large-scale expansion production, whereby up to 5 × 10^9^ TIL cells were harvested. These cells were infused back into patients after detecting the immunophenotyped TILs.

### 2.5. TIL Immunophenotyping

The cultured TIL phenotypes after culture were characterized using flow cytometry with anti-CD3 (Cat#: 555339, 1.5 *μ*L/10^6^ cells), anti-CD4 (Cat#: 557871, 2 *μ*L/10^6^ cells), anti-CD8 (Cat#: 563823, 2 *μ*L/10^6^ cells), anti-CD56 (Cat#: 56275, 3 *μ*L/10^6^ cells), and anti-PD1 (Cat#: 561272, 5 *μ*L/10^6^ cells) for 30 minutes on ice in the dark [35, 37]. Thereafter, the cells were washed once with PBS and resuspended in 400 *μ*L PBS. 7AAD was used to distinguish live cells and dead cells, and the cells were run on a BD Fortessa (BD Biosciences). Fluorescence minus one (FMO) was used as the negative control. Moreover, FlowJo software was used to analyze the data generated by flow cytometry. FoxP3 staining was conducted using an intracellular staining protocol from BD Biosciences as follows: anti-CD3 and anti-CD4 were stained for 30 minutes on ice in the dark; TILs were washed, fixed, and permeabilized following protocols for BD Fix Buffer I (Cat#: 557870, BD Biosciences, USA) and Perm Buffer III (Cat#: 558050, BD Biosciences, USA). The cells were washed thrice with Perm Buffer III and incubated with anti-FoxP3 (Cat#: 560460, 5 *μ*L/10^6^ cells) for 30 minutes on ice in the dark. The cells were run on a BD Fortessa (BD Biosciences). Fluorescence minus one (FMO) was used as the negative control. FlowJo software was used to analyze the data generated by flow cytometry.

### 2.6. Statistical Analysis

GraphPad Prism 7.0 and SPSS 17.0 software were used for statistical analysis. PFS and OS were calculated by Kaplan-Meier. OS and PFS were calculated from the start of TIL therapy. Univariable and multivariable Cox proportional hazards regression models were used to estimate hazard ratios along with associated confidence intervals and *p* values. Other data used *t*-test or *χ*^2^ test. For all statistical analyses, significance is indicated as at least *p* < 0.05.

## 3. Results

### 3.1. Patient Characteristics

Between June 2016 and March 2018, 60 patients with chemotherapy-resistant metastatic osteosarcoma were enrolled in this study, and they were treated with TILs and nivolumab therapy. Detailed characteristics of the patients are shown in Table 1.

### 3.2. Phenotype of TILs

**Figure 1:**
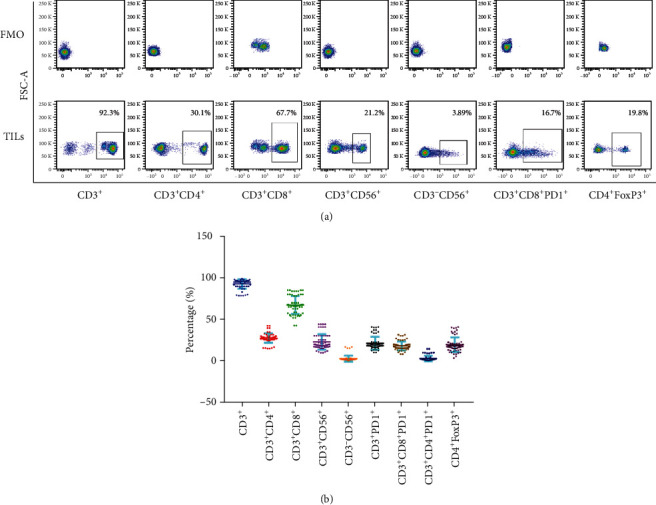
Phenotype of TILs at the time of infusion. (a) Representative flow cytometry of CD3^+^, CD3^+^CD4^+^, CD3^+^CD8^+^, CD3^+^CD56^+^, CD3^−^CD56^+^, CD3^+^CD8^+^PD1^+^, and CD4^+^FoxP3^+^percentage of TILs. (b) Quantitative analysis of CD3^+^, CD3^+^CD4^+^, CD3^+^CD8^+^, CD3^+^CD56^+^, CD3^−^CD56^+^, CD3^+^PD1^+^, CD3^+^CD8^+^PD1^+^, CD3^+^CD8^+^PD1^+^, and CD4^+^FoxP3^+^percentage of TILs. FMO is negative control.

The total number of TILs at infusion time was averagely 5 × 10^9^ cells (range, 3-8 × 10^9^). The TILs were primarily CD3^+^ T cells (92.84% ± 5.61%, *N* = 60) and comprised CD8^+^ T cells (67.55% ± 10.84%, *N* = 60), CD4^+^ T cells (27.87% ± 5.64%, *N* = 60), NK cells (3.14% ± 3.67%, *N* = 60), and NKT cells (23.21% ± 9.47%, *N* = 60). PD1 was expressed as the mean ± SD of 21.51% ± 7.85% of infused TILs, primarily on CD8^+^ T cells (18.31% ± 5.30%, *N* = 60). A subgroup of Foxp3^+^ T regulatory cells (19.75% ± 8.80%) was isolated from the CD3^+^CD4^+^ T cell population (Figure 1).

### 3.3. Treatment-Related Toxicities

The most common AEs of combined TILs and anti-PD1 therapy included fever, fatigue, rash, anorexia, leukopenia, and anemia (Table 2). All grades of treatment-associated AEs occurred in 45 patients (75%), and 43 of the 45 patients were grade 1 or 2 (95.56%). Grade 3 or 4 treatment-associated AEs were observed in two patients (3.33%). One patient exhibited a grade 4 fever during treatment; however, objective antitumor regression (complete response (CR)) was observed in this patient after 6 cycles of combined TILs and nivolumab therapy. Besides, grade 3 fever was observed in another patient with CR after 6 cycles of combined TILs and nivolumab therapy. Notably, fever was the most frequently observed AE, which occurred in 32 patients (53.33%). Nearly all fever cases rose no higher than 38°C and spontaneously resolved within 12 hours. The patients with grade 3 and 4 fever were treated with nonsteroidal anti-inflammatory drugs and resolved to a normal level within 48 hours. No patient exhibited other treatment-associated serious AEs. Moreover, infections, vitiligo, nausea, or vomiting was not observed following combined TILs and nivolumab therapy. No patient was discontinued from any treatment due to treatment-associated AEs.

### 3.4. Treatment Outcomes

**Figure 2:**
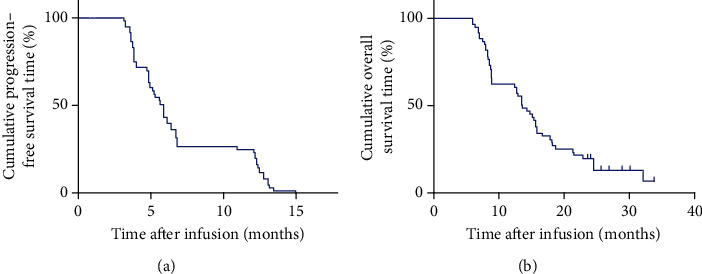
Kaplan-Meier curves for PFS and OS of patients, *N* = 60. (a) The PFS curve of patients. (b) The OS curve of patients.

**Figure 3:**
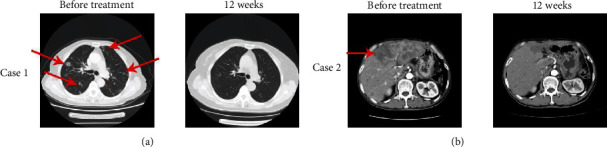
Two complete responses of patients with TIL therapy combined with anti-PD1 therapy. (a) A 15-year-old patient with lung metastasis experienced a complete response (CR) after 12 weeks of TIL therapy plus anti-PD1 therapy. The red arrow points at the target lesions before treatment. (b) A 20-year-old patient with liver metastasis experienced a complete response (CR) after 12 weeks of TIL therapy plus anti-PD1 therapy. The red arrow points at the target lesions before treatment.

**Figure 4:**
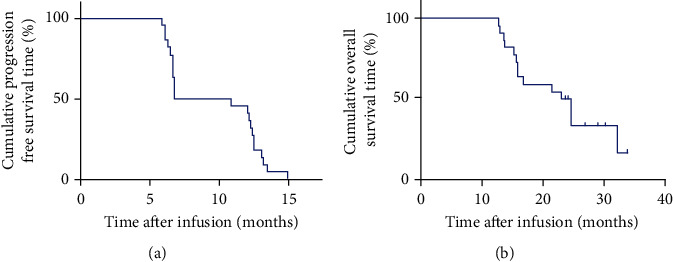
Kaplan-Meier curves for PFS and OS of patients with ORR (CR+PR), *N* = 22. (a) The PFS curve of patients with ORR. (b) The OS curve of patients with ORR.

The ORR was recorded in 22 out of 60 patients (36.67%) including 2 with a CR and 20 with a partial response (PR). The disease control rate (DCR) was observed in 48 patients (80%). During the last follow-up in February 2020, all the patients experienced disease progression, 50 patients had died, and 10 were alive. The mPFS and mOS were 5.75 and 13.6 months, respectively (Figures 2(a) and 2(b)). The 1-year PFS and OS rates were 25% (95% CI: 13%, 37%) and 60% (95% CI: 50%, 72%), respectively. Additionally, patients who experienced a CR were 15-year-old and 20-year-old males with lung metastases and liver metastases, respectively. After 12 weeks of combined TILs and nivolumab therapy, the multiple lung metastases (Figure 3(a)) and liver metastases (Figure 3(b)) disappeared. The PFS was 15 months and 12.1 months for the first patient and the second patient, respectively, and the two patients are so far alive. The 22 patients with CR+PR achieved an mPFS for 8.85 months (Figure 4(a)) and an mOS for 23.7 months (Figure 4(b)). Of note, 8 of the 20 patients with PR are currently alive for the last follow-up.

### 3.5. Characteristics of Patients with ORR

**Figure 5:**
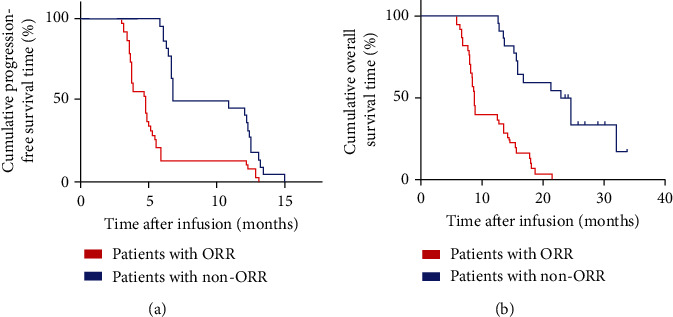
Kaplan-Meier curves for PFS and OS of patients with ORR (CR+PR), *N* = 22, and non-ORR, *N* = 38. (a) The PFS curve of patients with ORR and non-ORR. (b) The OS curve of patients with ORR and non-ORR. The blue line shows patients with ORR, and the red line shows patients with non-ORR.

The mPFS and mOS of the patients with ORR (*N* = 22) and patients with non-ORR (*N* = 38) were analyzed. The mPFS and mOS of ORR versus non-ORR were 8.85 months vs. 4.8 months (*p* < 0.0001) and 23.7 months vs. 8.7 months (*p* < 0.0001), respectively (Figures 5(a) and 5(b)). Notably, patients with ORR could highly benefit from combined TILs and anti-PD1 therapy. Therefore, we explored the biomarkers for this therapy based on the characteristics of patients with ORR. Many prognostic factors were reported for predicting osteosarcoma progression [38–41]. First, characteristics of patients with ORR and non-ORR patients were analyzed based on gender, ages, ECOG PS, site and size of the primary tumor, response to neoadjuvant chemotherapy, and location of metastasis. These factors are significant prognostic factors for patients with osteosarcoma; however, there were no significant differences between these factors in patients with ORR and non-ORR (Table 3). Interestingly, significant differences in the infusions of TIL numbers, CD8^+^TIL percentage, CD8^+^PD1^+^TIL percentage, and CD4^+^FoxP3^+^TIL percentage were reported between patients with ORR and non-ORR (Table 3). The infusion of TIL numbers and CD8^+^TIL percentage in patients with ORR versus patients with non-ORR was 6.2 × 10^9^ ± 1.1 × 10^9^ vs. 2.5 × 10^9^ ± 1.4 × 10^9^ (*p* < 0.0001) and 75.3% ± 3.2% vs. 52.2% ± 4.1% (*p* < 0.0001), respectively. Contrarily, infusion of CD8^+^PD1^+^TIL percentage and CD4^+^FoxP3^+^TIL percentage in patients with ORR versus patients with non-ORR was 5.1% ± 1.3% vs. 25.8% ± 3.1% (*p* < 0.0001) and 12.5% ± 3.6% vs. 24.0% ± 8.1% (*p* < 0.0001), respectively. Overall, more infusion of TIL numbers and CD8^+^TIL percentage, less infusion of CD8^+^PD1^+^TIL percentage, and CD4^+^FoxP3^+^TIL percentage are potentially significant factors for predicting response to combined TILs and anti-PD1 therapy.

### 3.6. Prognostic Factors of Combined TILs and Anti-PD1 Therapy

**Figure 6:**
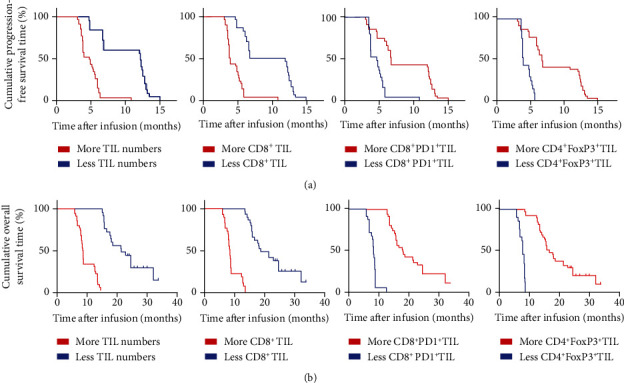
Univariate analyses of more infusion of TIL numbers and CD8^+^TIL percentage and less infusion of CD8^+^PD1^+^TIL percentage and CD4^+^FoxP3^+^TIL percentage based on PFS and OS. (a) The PFS curve of patients, A: patients with more TIL numbers (≥5 × 10^9^, blue line) and less TIL numbers (<5 × 10^9^, red line); B: patients with more CD8^+^TIL (≥60%, blue line) and less CD8^+^TIL (<60%, red line); C: patients with more CD8^+^PD1^+^TIL (≥10%, blue line) and less CD8^+^PD1^+^TIL (<10%, red line); D: patients with more CD4^+^FoxP3^+^TIL (≥20%, blue line) and less CD4^+^FoxP3^+^TIL (<20%, red line). (b) The OS curve of patients, A: patients with more TIL numbers (≥5 × 10^9^, blue line) and less TIL numbers (<5 × 10^9^, red line); B: patients with more CD8^+^TIL (≥60%, blue line) and less CD8^+^TIL (<60%, red line); C: patients with more CD8^+^PD1^+^TIL (≥10%, blue line) and less CD8^+^PD1^+^TIL (<10%, red line); D: patients with more CD4^+^FoxP3^+^TIL (≥20%, blue line) and less CD4^+^FoxP3^+^TIL (<20%, red line).

Patients with ORR had more infusion of TIL numbers and CD8^+^TIL percentage but less infusion of CD8^+^PD1^+^TIL percentage and CD4^+^FoxP3^+^TIL percentage. Therefore, potential prognostic factors that could predict clinical response to combined TILs and anti-PD1 therapy were assessed. There were no significant differences in mPFS and mOS based on gender, ages, ECOG PS, site and size of the primary tumor, response to neoadjuvant chemotherapy, and location of metastasis using Kaplan-Meier analysis (Table 4). Contrarily, univariate analyses proposed that more infusion of TIL numbers and CD8^+^TIL percentage and less infusion of CD8^+^PD1^+^TIL percentage and CD4^+^FoxP3^+^TIL percentage were significantly associated with increased mPFS (12.2 months vs. 4.8 months, *p* < 0.0001; 9.45 months vs. 3.85 months, *p* < 0.0001; 6.8 months vs. 4.8 months, *p* < 0.0001; and 6.7 months vs. 3.9 months, *p* < 0.0001) (Figure 6(a), A–D) and mOS (21.4 months vs. 8.6 months, *p* < 0.0001; 18.7 months vs. 8.4 months, *p* < 0.0001; 18.0 months vs. 8.2 months, *p* < 0.0001; and 16.7 months vs. 7.9 months, *p* < 0.0001) (Figure 6(b), A–D). These differences were significant in the multivariate Cox proportional hazards model (*p* < 0.0001) in mPFS (Table 5) and mOS (Table 6). Conclusively, more infusion of TIL numbers and CD8^+^TIL percentage and less infusion of CD8^+^PD1^+^TIL percentage and CD4^+^FoxP3^+^TIL percentage may be potential prognostic factors which can predict clinical response to combined TILs and anti-PD1 therapy.

## 4. Discussion

Immunotherapy has improved the field of oncology and is largely attributed to the success of immune checkpoint inhibitors. However, the durability and efficacy of anti-PD1 therapy vary across different malignancies. Many studies have been conducted on the use of anti-PD1 against osteosarcoma; however, the ORR of nonselective patients is less than 10% which significantly lowers the effectiveness of anti-PD1 therapy to osteosarcoma [11]. The absence of TILs in the tumor microenvironment is one of the potential causes of tumor resistance to this immune checkpoint therapy [42]. Notably, TIL therapy has achieved successful clinical efficacy in treating melanoma since its first report by Rosenberg and colleagues more than 20 years ago [23]. The encouraging success achieved in TIL treatment against melanoma has stimulated scientists globally to conduct studies on other solid tumors, such as renal cell carcinoma, cervical cancer, and other epithelial cancers [27–30]. However, the clinical response of TIL therapy to these tumors is lower when compared to melanoma in general. Of note, there are limited studies of TILs on osteosarcoma, except for a previously conducted preclinical study [31]. In this study, benefits are derived from combined TILs and anti-PD1 therapy in treating chemotherapy-resistant metastatic osteosarcoma. Interestingly, this new treatment strategy displayed a promising antitumor effect and a satisfactory objective response with 22 out of the 60 patients exhibiting clinical tumor regression.

Moreover, inhibiting the PD1/PDL1 pathway released the brake on T lymphocytes and restored antitumor immune response resulting in tumor elimination [43]. Notably, a subpopulation of PD1^+^T lymphocytes was observed in the cultured TILs, suggesting that a PD1 blockade may significantly increase the cytotoxicity of TILs. Similarly, recent studies have reported that blocking the PD1 pathway significantly increased the antitumor effects of adoptive T lymphocyte immunotherapy performed with chimeric antigen receptor (CAR) T cells [44, 45]. Univariate and multivariate analyses indicated that patients with less infusion of CD8^+^PD1^+^TIL percentage showed better PFS and OS. Therefore, it was proposed that combined TILs and anti-PD1 therapy potentially increases clinical response rates and survival time in chemotherapy-resistant metastatic osteosarcoma.

In addition, effective treatment methods for metastatic osteosarcoma patients with chemotherapy resistance are unavailable. Therefore, exploring treatment methods for these patients is urgently significant. This study reported the efficacy of the combined TILs and anti-PD1 therapy in metastatic osteosarcoma patients exhibiting chemotherapy resistance. Out of the 60 patients, 22 patients showed an objective response, 2 with CR and 20 with PR. The mPFS was 5.75 months, whereas the mOS was 13.6 months. However, there is a sizable arsenal of chemotherapy agents with proven efficacy against osteosarcoma patients, and the mOS is no more than 6 months in metastatic osteosarcoma patients with chemotherapy resistance [46]. Therefore, combined TILs and anti-PD1 therapy may provide an improved treatment method for metastatic osteosarcoma patients exhibiting resistance to chemotherapy.

Many studies have confirmed that T cell infiltration in tumors is predictive of the OS of patients, indicating that T cells can restrict tumor growth [47–52]. However, most infiltrated tumors progress, suggesting that spontaneous antitumor immune responses are insufficient in controlling tumors. Furthermore, immune checkpoint inhibitors used as cancer therapies reverse T cell tolerance and mediate a proliferative response of TILs by blocking inhibitory interactions between tumor cells and infiltrating T cells, thus allowing for an antitumor immune response. However, the origin of this response has not been established because chronic activation promotes terminal differentiation or exhaustion of tumor-specific T cells [49]. Immunotherapies are aimed at boosting antitumor immune responses to induce durable tumor control. Current regimens mainly include adoptive cell therapy (“immune accelerator”) and checkpoint inhibitors (“immune brake”) which have yielded unprecedented clinical benefit in several tumor types. Besides, the efficacy of a single anti-PD1 against osteosarcoma is limited for nonselective patients [11]. Of note, TILs showed therapeutic efficacy against osteosarcoma in preclinical mouse models [31]. Synergism from this combination may be considered as ex vivo grown. Moreover, expanded tumor-reactive TILs are often PD1-positive; therefore, preventing the interaction between PD1 on T cells and PDL1 on tumor cells through anti-PD1 therapy during TIL infusion may render the TILs more tumoricidal [53]. Combined TILs and anti-PD1 therapy may increase the clinical benefits of osteosarcoma. In this study, the ORR of all the patients was 36.67% which is significantly higher than a single anti-PD1 therapy against osteosarcoma; this is consistent with reports from previous studies on other solid tumors [54, 55]. Besides, patients with less infusion of CD4^+^FoxP3^+^TIL percentage were reported to have better PFS and OS. Similarly, patients with more infusion of TIL numbers and CD8^+^TIL percentage showed better PFS and OS. The average numbers of infused TILs were approximately 5 × 10^9^, which is less than those reported from other studies in melanoma [25, 54]. Most of the melanoma patients received TIL immunotherapy. However, this study used combined TILs and anti-PD1 therapy to treat osteosarcoma patients; this may illustrate why the lower numbers of infused TILs can yield satisfactory efficacy. PDL1 expression has been correlated with higher response rates in several tumors, while osteosarcoma has been shown to have variable PDL1 expression and responses also seen even in the absence of PDL1 expression in several tumors [11, 56]. In the future, new studies showed be administered to help to elucidate the role of PDL1 in the treatment of combined TILs and anti-PD1 therapy. This observation should, therefore, be replicated in other future studies to determine whether characteristics of cultured TILs may truly represent the first biomarker predictive of response to this combined immunotherapy. Notably, the patients most likely to respond to treatment can eventually be identified.

Additionally, combined TILs and anti-PD1 therapy was well tolerated without an increase in serious adverse effects. This is different from previous study reports whereby TIL treatment yielded more adverse effects because of the lymphodepleting preparative regimens and the subsequent IL-2 after TIL infusion [16, 23, 24, 26]. In this study, patients only received transfusion of TILs combined with anti-PD1 therapy. Therefore, this treatment strategy was confirmed not to increase adverse effects. Two patients showed grade 3 or 4 fever and were treated with nonsteroidal anti-inflammatory drugs which resolved the fever within 48 hours. Interestingly, the two patients exhibited a CR. A study published in the *Journal of Clinical Oncology* showed that fever after anti-PD1 therapy may be an early predictor of response to anti-PD1 treatment [57]. Future studies should focus on exploring the association between fever and immunotherapy.

There are no current reports on the efficacy of combined TILs and anti-PD1 therapy against osteosarcoma. Besides, treatment with anti-CTLA-4 antibody ipilimumab has been shown to increase T cell infiltration into melanomas and broaden the TIL response to tumors. A clinical trial report indicated that 13 patients with metastatic melanoma were treated with ipilimumab and TIL therapy; notably, 5/13 of patients (38.5%) showed an ORR [58]. However, this study provides the first report that demonstrates the feasibility of combining TILs with immune checkpoint inhibitors. The role of TILs in combination with anti-PD1 is currently subject to clinical trials in treating melanoma (NCT03374839, NCT03475134, NCT03158935, NCT02652455, NCT02621021, and NCT01993719). However, whether TILs should be administered in combination with anti-PD1 or as a single treatment option is still unknown for solid tumors. The success in combined treatment for metastatic osteosarcoma patients exhibiting chemotherapy resistance confirms that TIL combination with anti-PD1 therapy may be a better treatment method for osteosarcoma. Therefore, in-depth studies need to be conducted in the future.

Conclusively, this study provides a report on 60 patients with chemotherapy-resistant metastatic osteosarcoma who received TIL therapy combined with anti-PD1 therapy. Although it is a single-center, nonrandomized retrospective study, this study can be an exploration of treatment for metastatic osteosarcoma and provides some significant clinical implications. Prospective randomized studies are worthy to apply to determine whether patients can achieve benefit from combined TILs with anti-PD1 therapy.

## Figures and Tables

**Table 1:** Patient characteristics (*N* = 60).

Characteristic	No. of patients	%
Gender		
Male	40	66.7
Female	20	33.3
Age (years)		
≥20	18	30
<20	42	70
ECOG PS		
0	44	73.3
1	16	26.7
Site of primary tumor		
Femur and tibia	38	63.3
Other	22	36.7
Size of primary tumor (cm)		
≥5	48	80
<5	12	20
Response to neoadjuvant chemotherapy		
Good	10	16.7
Poor	50	83.3
Location of metastasis		
Lung	50	83.3
Others	10	16.7

**Table 2:** Treatment-related adverse events in patients in response to therapy (*N* = 60).

Side effects	No. (%) of patients associated with adverse events	
	Grade 1 or 2	Grade 3 or 4
Fever	30 (50)	2 (3.33)
Fatigue	15 (25)	0
Rash	11 (18.33)	0
Anorexia	13 (21.67)	0
Leukopenia	9 (15)	0
Anemia	8 (13.33)	0
Vitiligo	0	0
Nausea	0	0
Vomiting	0	0
Total incidence	43 (71.67)	2 (3.33)

**Table 3:** Characteristics of patients with ORR (*N* = 22) and non-ORR (*N* = 38).

Characteristic	No. of ORR	No. of non-ORR	*p* value
Gender			
Male	14	26	
Female	8	12	0.705
Age (years)			
≥20	6	12	
<20	16	26	0.726
ECOG PS			
0	20	30	
1	2	8	0.231
Site of primary tumor			
Femur and tibia	12	26	
Other	10	12	0.282
Size of primary tumor (cm)			
≥5	17	31	
<5	5	7	0.688
Response to neoadjuvant chemotherapy			
Good	3	7	
Poor	19	31	0.632
Location of metastasis			
Lung	18	32	
Others	4	6	0.811
Infusion of TIL numbers			
≥5 × 10^9^	20	15	
<5 × 10^9^	2	23	0.000
Infusion of CD8^+^TIL percentage			
≥60%	19	11	
<60%	3	27	0.000
Infusion of CD8^+^PD1^+^TIL percentage			
≥10%	4	21	
<10%	18	17	0.005
Infusion of CD4^+^FoxP3^+^TIL percentage			
≥20%	2	19	
<20%	20	19	0.001

**Table 4:** Univariate analysis of factors related to mDFS and mOS of patients in this study (*N* = 60).

Characteristics	mDFS (months)	*p* value	mOS (months)	*p* value
Gender				
Male	5.75		13.5	
Female	5.55	0.838	15.5	0.111
Age (years)				
≥20	5.25		12.9	
<20	5.9	0.375	14.5	0.518
ECOG PS				
0	5.75		14.2	
1	5.75	0.496	12.9	0.985
Site of primary tumor				
Femur and tibia	5.75		13.5	
Other	5.6	0.425	15.3	0.424
Size of primary tumor (cm)				
≥5	5.55		13.6	
<5	6.2	0.87	14.0	0.668
Response to neoadjuvant chemotherapy				
Good	5.9		14.5	
Poor	5.2	0.242	13.5	0.566
Location of metastasis				
Lung	5.6		15.1	
Others	6.0	0.482	13.6	0.167

**Table 5:** Multivariate analysis (mPFS).

Parameters	Hazard ratio	95% confidence interval	*p* value
Infusion of CD8^+^TIL numbers (≥5 × 10^9^ vs. <5 × 10^9^)	3.73	(2.11, 6.57)	<0.0001
Infusion of CD8^+^TIL percentage (≥60% vs. <60%)	4.05	(2.15, 7.64)	<0.0001
Infusion of CD8^+^PD1^+^TIL percentage (≥10% vs. <10%)	2.98	(1.57, 5.66)	<0.0001
Infusion of CD4^+^FoxP3^+^TIL percentage (≥20% vs. <20%)	3.12	(1.98, 5.78)	<0.0001

**Table 6:** Multivariate analysis (mOS).

Parameters	Hazard ratio	95% confidence interval	*p* value
Infusion of CD8^+^TIL numbers (≥5 × 10^9^ vs. <5 × 10^9^)	5.30	(2.80, 10.03)	<0.0001
Infusion of CD8^+^TIL percentage (≥60% vs. <60%)	5.88	(2.85, 12.14)	<0.0001
Infusion of CD8^+^PD1^+^TIL percentage (≥10% vs. <10%)	6.38	(2.70, 15.08)	<0.0001
Infusion of CD4^+^FoxP3^+^TIL percentage (≥20% vs. <20%)	4.87	(2.56, 9.84)	<0.0001

## Data Availability

The data used to support the findings of this study are included within the article.

## References

[1] Siegel R. L., Miller K. D., Jemal A. (2018). Cancer statistics, 2019. *CA: A Cancer Journal for Clinicians*.

[2] Carrle D., Bielack S. (2009). Osteosarcoma lung metastases detection and principles of multimodal therapy. *Cancer Treatment and Research*.

[3] Strauss S. J., Ng T., Mendoza-Naranjo A., Whelan J., Sorensen P. H. B. (2010). Understanding micrometastatic disease and anoikis resistance in Ewing family of tumors and osteosarcoma. *The Oncologist*.

[4] Provisor A. J., Ettinger L. J., Nachman J. B. (1997). Treatment of nonmetastatic osteosarcoma of the extremity with preoperative and postoperative chemotherapy: a report from the Children’s Cancer Group. *Journal of Clinical Oncology*.

[5] Antman K., Crowley J., Balcerzak S. P. (1998). A southwest oncology group and cancer-and leukemia group B phase II study of doxorubicin, dacarbazine, ifosfamide, and mesna in adults with advanced osteosarcoma, Ewing’s sarcoma, and rhabdomyosarcoma. *Cancer*.

[6] Zalupski M. M., Rankin C., Ryan J. R. (2004). Adjuvant therapy of osteosarcoma - a phase II trial: Southwest Oncology Group study 9139. *Cancer*.

[7] Bielack S. S., Hecker-Nolting S., Blattmann C., Kager L. (2016). Advances in the management of osteosarcoma. *F1000Research*.

[8] Tsuchiya H., Kanazawa Y., Abdel-Wanis M. E. (2002). Effect of timing of pulmonary metastases identification on prognosis of patients with osteosarcoma: the Japanese Musculoskeletal Oncology Group study. *Journal of Clinical Oncology*.

[9] Kager L., Zoubek A., Pötschger U. (2003). Primary metastatic osteosarcoma: presentation and outcome of patients treated on neoadjuvant Cooperative Osteosarcoma Study Group protocols. *Journal of Clinical Oncology*.

[10] Antonia S. J., López-Martin J. A., Bendell J. (2016). Nivolumab alone and nivolumab plus ipilimumab in recurrent small-cell lung cancer (CheckMate 032): a multicentre, open-label, phase 1/2 trial. *The Lancet Oncology*.

[11] Tawbi H. A., Burgess M., Bolejack V. (2017). Pembrolizumab in advanced soft-tissue sarcoma and bone sarcoma (SARC028): a multicentre, two-cohort, single-arm, open-label, phase 2 trial. *The Lancet Oncology*.

[12] Gandhi L., Rodríguez-Abreu D., Gadgeel S. (2018). Pembrolizumab plus chemotherapy in metastatic non-small-cell lung cancer. *The New England Journal of Medicine*.

[13] Rini B. I., Plimack E. R., Stus V. (2019). Pembrolizumab plus axitinib versus sunitinib for advanced renal-cell carcinoma. *The New England Journal of Medicine*.

[14] Hellmann M. D., Paz-Ares L., Caro R. B. (2019). Nivolumab plus ipilimumab in advanced non-small-cell lung cancer. *The New England Journal of Medicine*.

[15] News Release F (2017). FDA approves first cancer treatment for any solid tumor with a specific genetic feature. *Molecular and Cellular Pharmacology*.

[16] Bernhard H., Neudorfer J., Gebhard K. (2008). Adoptive transfer of autologous, HER2-specific, cytotoxic T lymphocytes for the treatment of HER2-overexpressing breast cancer. *Cancer Immunology, Immunotherapy*.

[17] Gros A., Robbins P. F., Yao X. (2014). PD-1 identifies the patient-specific CD8+ tumor-reactive repertoire infiltrating human tumors. *The Journal of Clinical Investigation*.

[18] Schell T. D., Knowles B. B., Tevethia S. S. (2000). Sequential loss of cytotoxic T lymphocyte responses to simian virus 40 large T antigen epitopes in t antigen transgenic mice developing osteosarcomas. *Cancer Research*.

[19] Lussier D. M., O’Neill L., Nieves L. M. (2015). Enhanced T-cell immunity to osteosarcoma through antibody blockade of PD-1/PD-L1 interactions. *Journal of Immunotherapy*.

[20] Ichino Y., Ishikawa T. (1983). Cytolysis of autologous fresh osteosarcoma cells by human cytotoxic T lymphocytes propagated with T cell growth factor. *GANN Japanese Journal of Cancer Research*.

[21] Tumeh P. C., Harview C. L., Yearley J. H. (2014). PD-1 blockade induces responses by inhibiting adaptive immune resistance. *Nature*.

[22] Schumacher T. N., Schreiber R. D. (2015). Neoantigens in cancer immunotherapy. *Science*.

[23] Rosenberg S. A., Yannelli J. R., Yang J. C. (1994). Treatment of patients with metastatic melanoma with autologous tumor-infiltrating lymphocytes and interleukin 2. *Journal of the National Cancer Institute*.

[24] Dudley M. E., Yang J. C., Sherry R. (2008). Adoptive cell therapy for patients with metastatic melanoma: evaluation of intensive myeloablative chemoradiation preparative regimens. *Journal of Clinical Oncology*.

[25] Besser M. J., Shapira-Frommer R., Itzhaki O. (2013). Adoptive transfer of tumor-infiltrating lymphocytes in patients with metastatic melanoma: intent-to-treat analysis and efficacy after failure to prior immunotherapies. *Clinical Cancer Research*.

[26] Andersen R., Donia M., Ellebaek E. (2016). Long-lasting complete responses in patients with metastatic melanoma after adoptive cell therapy with tumor-infiltrating lymphocytes and an attenuated il 2 regimen. *Clinical Cancer Research*.

[27] Yannelli J. R., Hyatt C., McConnell S. (1996). Growth of tumor-infiltrating lymphocytes from human solid cancers: summary of a 5-year experience. *International Journal of Cancer*.

[28] Stevanović S., Draper L. M., Langhan M. M. (2015). Complete regression of metastatic cervical cancer after treatment with human papillomavirus-targeted tumor-infiltrating T cells. *Journal of Clinical Oncology*.

[29] Andersen R., Westergaard M. C. W., Kjeldsen J. W. (2018). T-cell responses in the microenvironment of primary renal cell carcinoma-implications for adoptive cell therapy. *Cancer Immunology Research*.

[30] Stevanović S., Helman S. R., Wunderlich J. R. (2019). A phase II study of tumor-infiltrating lymphocyte therapy for human papillomavirus–associated epithelial cancers. *Clinical Cancer Research*.

[31] Théoleyre S., Mori K., Cherrier B. (2005). Phenotypic and functional analysis of lymphocytes infiltrating osteolytic tumors: use as a possible therapeutic approach of osteosarcoma. *BMC Cancer*.

[32] Chen C.-L., Pan Q.-Z., Weng D.-S. (2017). Safety and activity of PD-1 blockade-activated DC-CIK cells in patients with advanced solid tumors. *OncoImmunology*.

[33] Schwartz L. H., Litière S., de Vries E. (2016). RECIST 1.1- update and clarification: from the RECIST committee. *European Journal of Cancer*.

[34] Jun G. T., Ward J., Clarkson P. J. (2010). Systems modelling approaches to the design of safe healthcare delivery: ease of use and usefulness perceived by healthcare workers. *Ergonomics*.

[35] Li W., Xu L., Wang Y., Zhao L., Kellner D. B., Gao Q. (2017). Efficacy of tumor-infiltrating lymphocytes combined with IFN-*α* in Chinese resected stage III malignant melanoma. *Journal of Immunology Research*.

[36] Yin H., Guo W., Sun X., Li R., Feng C., Tan Y. (2020). TILs and anti-PD1 therapy: an alternative combination therapy for PDL1 negative metastatic cervical cancer. *Journal of Immunology Research*.

[37] Lu Y., Guo L., Ding G. (2019). PD1+ tumor associated macrophages predict poor prognosis of locally advanced esophageal squamous cell carcinoma. *Future Oncology*.

[38] Bielack S. S., Kempf-Bielack B., Delling G. (2002). Prognostic factors in high-grade osteosarcoma of the extremities or trunk: an analysis of 1, 702 patients treated on neoadjuvant cooperative osteosarcoma study group protocols. *Journal of Clinical Oncology*.

[39] Bacci G., Longhi A., Versari M., Mercuri M., Briccoli A., Picci P. (2006). Prognostic factors for osteosarcoma of the extremity treated with neoadjuvant chemotherapy. *Cancer*.

[40] Whelan J. S., Jinks R. C., McTiernan A. (2012). Survival from high-grade localised extremity osteosarcoma: combined results and prognostic factors from three European osteosarcoma intergroup randomised controlled trials. *Annals of Oncology*.

[41] Bertrand T. E., Cruz A., Binitie O., Cheong D., Letson G. D. (2016). Do surgical margins affect local recurrence and survival in extremity, nonmetastatic, high-grade osteosarcoma?. *Clinical Orthopaedics and Related Research*.

[42] Zhang J., Li Y., Yang S., Zhang L., Wang W. (2019). Anti-CD40 mAb enhanced efficacy of anti-PD1 against osteosarcoma. *Journal of Bone Oncology*.

[43] Taube J. M., Klein A., Brahmer J. R. (2014). Association of PD-1, PD-1 ligands, and other features of the tumor immune microenvironment with response to anti-PD-1 therapy. *Clinical Cancer Research*.

[44] John L. B., Devaud C., Duong C. P. M. (2013). Anti-PD-1 antibody therapy potently enhances the eradication of established tumors by gene-modified T cells. *Clinical Cancer Research*.

[45] Chong E. A., Melenhorst J. J., Lacey S. F. (2017). PD-1 blockade modulates chimeric antigen receptor (CAR)-modified T cells: refueling the CAR. *Blood*.

[46] Chou A. J., Gorlick R. (2014). Chemotherapy resistance in osteosarcoma: current challenges and future directions. *Expert Review of Anticancer Therapy*.

[47] Gettinger S. N., Choi J., Mani N. (2018). A dormant TIL phenotype defines non-small cell lung carcinomas sensitive to immune checkpoint blockers. *Nature Communications*.

[48] Wong P. F., Wei W., Smithy J. W. (2019). Multiplex quantitative analysis of tumor-infiltrating lymphocytes and immunotherapy outcome in metastatic melanoma. *Clinical Cancer Research*.

[49] Siddiqui I., Schaeuble K., Chennupati V. (2019). Intratumoral Tcf 1 + PD-1 + CD8 + T cells with stem-like properties promote tumor control in response to vaccination and checkpoint blockade immunotherapy. *Immunity*.

[50] Sundara Y. T., Kostine M., Cleven A. H. G., Bovée J. V. M. G., Schilham M. W., Cleton-Jansen A. M. (2017). Increased PD-L1 and T-cell infiltration in the presence of HLA class I expression in metastatic high-grade osteosarcoma: a rationale for T-cell-based immunotherapy. *Cancer Immunology, Immunotherapy*.

[51] Gomez-Brouchet A., Illac C., Gilhodes J. (2017). CD163-positive tumor-associated macrophages and CD8-positive cytotoxic lymphocytes are powerful diagnostic markers for the therapeutic stratification of osteosarcoma patients: an immunohistochemical analysis of the biopsies fromthe French OS2006 phase 3 trial. *Oncoimmunology*.

[52] Yunger S., El A. B., Zeltzer L.-a. (2019). Tumor-infiltrating lymphocytes from human prostate tumors reveal anti-tumor reactivity and potential for adoptive cell therapy. *Oncoimmunology*.

[53] Donia M., Kjeldsen J. W., Andersen R. (2017). PD-1+polyfunctional T cells dominate the periphery after tumor-infiltrating lymphocyte therapy for cancer. *Clinical Cancer Research*.

[54] Saint-Jean M., Knol A.-C., Volteau C. (2018). Adoptive cell therapy with tumor-infiltrating lymphocytes in advanced melanoma patients. *Journal of Immunology Research*.

[55] Yao X., Ahmadzadeh M., Lu Y. C. (2012). Levels of peripheral CD4+FoxP3+ regulatory T cells are negatively associated with clinical response to adoptive immunotherapy of human cancer. *Blood*.

[56] Hamid O., Robert C., Daud A. (2013). Safety and tumor responses with lambrolizumab (anti-PD-1) in melanoma. *The New England Journal of Medicine*.

[57] Chen S.-C. (2018). Fever after anti-programmed cell death-1 treatment to predict the response in advanced hepatocellular carcinoma. *Journal of Clinical Oncology*.

[58] Mullinax J. E., Hall M. L., Prabhakaran S. (2018). Combination of ipilimumab and adoptive cell therapy with tumor-infiltrating lymphocytes for patients with metastatic melanoma. *Frontiers in Oncology*.

